# Members of the genus *Burkholderia*: good and bad guys

**DOI:** 10.12688/f1000research.8221.1

**Published:** 2016-05-26

**Authors:** Leo Eberl, Peter Vandamme

**Affiliations:** 1 Department of Plant and Microbial Biology, University Zürich, Zurich, CH-8008, Switzerland; 2 Laboratory of Microbiology, Ghent University, Ledeganckstraat 35, B-9000 Gent, Belgium

## Abstract

In the 1990s several biocontrol agents on that contained *Burkholderia* strains were registered by the United States Environmental Protection Agency (EPA). After risk assessment these products were withdrawn from the market and a moratorium was placed on the registration of *Burkholderia*-containing products, as these strains may pose a risk to human health. However, over the past few years the number of novel *Burkholderia* species that exhibit plant-beneficial properties and are normally not isolated from infected patients has increased tremendously. In this commentary we wish to summarize recent efforts that aim at discerning pathogenic from beneficial *Burkholderia* strains.

## The genus *Burkholderia*: past and present

When the genus *Burkholderia* was defined in 1992 by Yabuuchi *et al.* to accommodate most of the former rRNA group II pseudomonads, it consisted of only seven species ^1^. Two of these species ( *Burkholderia pseudomallei* and *Burkholderia mallei*) are primary pathogens of animals and humans, two species ( *Burkholderia caryophylli* and *Burkholderia gladioli*) are known as plant pathogens, two species ( *Burkholderia solanacearum* [a plant pathogen] and *Burkholderia pickettii* [an opportunistic human pathogen]) were later transferred to the genus *Ralstonia,* and the remaining species, *Burkholderia cepacia,* was originally described as the causative agent of bacterial rot of onion bulbs ^2^. Since the first description of the genus, the number of validly named species has increased to almost one hundred ( http://www.bacterio.net/burkholderia.html). During this time, it has become apparent that this genus has tremendous biotechnological potential, with species that produce a large variety of commercially important hydrolytic enzymes and bioactive substances, that promote plant growth and health, and that can degrade various recalcitrant pollutants. Yet their agricultural and industrial use is severely limited due to the potential threat that some strains pose to human health ^3^. In addition to *B. pseudomallei* and *B. mallei*, it is a group of currently 20 closely related bacterial species in particular, referred to as the *Burkholderia cepacia* complex (Bcc), which have emerged as opportunistic pathogens that can cause severe infections in cystic fibrosis (CF) and immunocompromised patients ^4– 6^. However, virtually all Bcc species have also been isolated from the natural environment, often from soil samples or from the rhizosphere of various plants. The use of *Burkholderia* in agricultural applications is therefore considered a double-edged sword, and a lot of effort has been invested into discriminating between the beneficial environmental (the good) and the clinical (the bad) *Burkholderia* strains ^7, 8^. Recently, these efforts have gained momentum, as many new *Burkholderia* species have been identified in environmental samples that exhibit potentially valuable beneficial traits. These species are believed to be safe for applications, as there are very rarely clinical reports that they would pose a risk to human health.

## *Burkholderia* species in the environment

Recent work has shown that members of the genus *Burkholderia* are common soil inhabitants and that their biogeographic distribution is strongly affected by soil pH ^9– 12^. Due to their intrinsic acid tolerance, *Burkholderia* strains have a competitive advantage in acidic soils but are outcompeted in alkaline soils. Moreover, it has been reported that *Burkholderia* significantly co-occurs with a wide range of fungi, which normally also prefer acidic environments ^13^. This finding is in line with reports demonstrating that many *Burkholderia* species can form either antagonistic or mutualistic interactions with fungi. While antagonistic behavior of *Burkholderia* species is well described and is dependent on the production of a large variety of antifungal compounds (for a review, see 14), other species have been demonstrated to live in mutualistic associations with fungi. A well-investigated example is represented by the association of *Burkholderia terrae* and *Lyophyllum* species, for which it was shown that the bacteria can not only use the hyphae of the fungus for transportation and dispersal but also use fungal exudates as nutrients ^15– 17^. This is in full agreement with the finding that *Burkholderia* strains are among the main consumers of carbon released from arbuscular mycorrhizal fungi ^18^. Another intriguing example is *Burkholderia rhizoxinica,* which invades hyphae of the fungus *Rhizopus microsporus* ^19, 20^, the causative agent of rice seedling blight. The symbiont is involved in the biosynthesis of the antimitotic toxin rhizoxin ^21^, which efficiently stalls plant cell division. In the absence of the endosymbiont, the fungus was found to be unable to reproduce vegetatively ^22^.

Another emerging theme is the tight association of some *Burkholderia* species with plants. Over the past few years, the number of novel plant-associated *Burkholderia* species has increased tremendously. These new species show various degrees of plant dependence, with some strains living freely in the rhizosphere, exhibiting an endophytic lifestyle, nodulating legumes, or, most intriguingly, forming an obligate leaf symbiosis with their host plants. *Burkholderia* species have been frequently isolated from diverse surface-sterilized plants (e.g. 23– 27). Probably the best studied endophytic *Burkholderia* strain is *Burkholderia phytofirmans* PsJN, which was originally isolated from onion roots and was subsequently demonstrated to establish endophytic populations in various plants ^28, 29^. Interestingly, *B. phytofirmans* is not only capable of protecting plants from pathogens (through an unknown mechanism) but was also shown to increase the plants’ stress resistance, particularly against low temperatures, high salt, and drought ^30– 32^. Some *Burkholderia* species have been shown to be specifically associated with *Sphagnum* mosses ^33, 34^. Since Moulin *et al*. demonstrated that two *Burkholderia* species, which were isolated from root nodules of a legume, possessed nodulation genes ^35^, many more nodulating *Burkholderia* species have been described (for recent studies, see 36– 38). Although these strains have mainly been isolated from *Mimosa* species, recent work showed that some *Burkholderia* strains can also nodulate fynbos legumes in South Africa ^39– 43^. Some plant genera of the *Rubiaceae* and *Primulaceae* families carry members of the genus *Burkholderia* within leaf nodules ^44– 47^. This unique association is the only known example of an obligate plant-bacterium symbiosis with both partners being unable to exist outside the symbiotic association. The bacterial symbiont is thought to be hereditarily transmitted to the progeny *via* colonization of the developing seeds. Although the molecular nature of the leaf nodule symbiosis is still unknown, it was recently shown that the bacterial symbiont produces large amounts of secondary metabolites, which appear to protect the plants from herbivores ^48, 49^.

Finally, a large body of evidence demonstrates that many insect species harbor symbiotic bacteria of the genus *Burkholderia* ^50– 53^. The association of *Burkholderia* species with the bean bug *Riptortus pedestris* has emerged as a promising experimental model to study the molecular mechanisms involved in insect-bacterium symbiosis ^54, 55^. This symbiosis appears to be particularly tight, as it was recently reported that the insect has a previously unrecognized animal organ used to specifically sort the symbiont into the posterior gut region, which is devoid of food flow and is transformed into an isolated organ for symbiosis ^56^.

We are convinced that these examples represent just the tip of the iceberg and that many more *Burkholderia* fungus/plant/insect associations will be discovered in the future.

## Can we tell the good from the bad by taxonomy?

**Figure 1.**
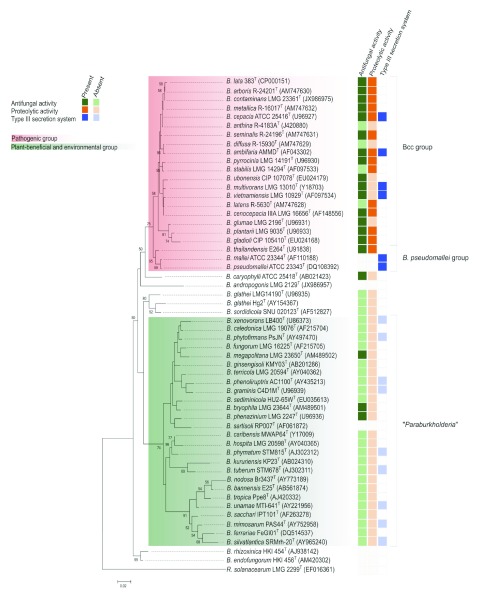
Maximum-likelihood phylogenetic reconstruction based on 16S rRNA gene sequences of 55 *Burkholderia* species and *Ralstonia solanacearum* LMG 2299T (outgroup). The alignment was performed using SINA v1.2.11 ( http://www.arb-silva.de/aligner/) ^93^. After gap removal with TrimAl ^94^, the final alignment consisted of 1289 positions. The phylogenetic reconstruction was conducted with MEGA6 ^95^ using Tamura-Nei evolutionary model ^96^ with gamma rate distribution (five gamma categories and 70% of invariable sites). Bootstrap test values are shown if greater than 50%. Some phenotypic characteristics are indicated. No boxes indicate that no information is available. The information on the presence of the type III secretion system is taken from 73.

Phylogenetic investigations have provided evidence that members of the genus *Burkholderia* can be divided into two main lineages ( Figure 1) and several species that represent unique lines of descent. One clade comprises pathogens of humans, animals, and plants, including *B. pseudomallei, B. mallei*, and *Burkholderia glumae*, as well as the Bcc species. However, this clade also contains many strains that can be used for plant growth promotion and biocontrol of plant pests, including *Burkholderia vietnamiensis* TVV74 and *Burkholderia ambifaria* AMMD, respectively ^57^. Ironically, although *Burkholderia cenocepacia* is generally considered the most problematic Bcc species in patients with CF ^58^, recently a genome sequence of a plant-beneficial endophytic *B. cenocepacia* strain with both biocontrol and plant-growth-promoting characteristics was reported ^59^. Also, non-Bcc *Burkholderia* species within this clade can have both beneficial and harmful properties. One intriguing case with great potential for agricultural applications is represented by *Burkholderia gladioli*, which is a well-known pathogen of plants (e.g. causing rice panicle blight) ^60^ as well as humans ^61– 63^. However, recent work has demonstrated that some *B. gladioli* strains live endophytically within various wild and ancient *Zea* plants without causing any disease symptoms ^64, 65^. In contrast, this endophyte was shown to produce an unidentified antifungal compound *in planta* and was able to suppress the fungal pathogen *Sclerotinia homoeocarpa* ^66^.

The second main phylogenetic *Burkholderia* cluster contains many plant-beneficial environmental *Burkholderia* species, as mentioned above ^67^. Several of these species have been reported to fix nitrogen, to be capable of nodulating legumes, to promote plant growth, and to degrade recalcitrant compounds ^68^. Given that species of this cluster are only rarely isolated from infected patients ^69– 71^, they are often considered to pose no risk to human health and have therefore been suggested to be promising candidates for applications in biocontrol, biofertilization, and bioremediation ^72– 74^. In our opinion, this is a wishful, potentially dangerous, and certainly oversimplified view.

Dividing the genus *Burkholderia* into two genera was recently proposed, with the novel genus *Paraburkholderia* containing the primarily environmental species (the alleged good ones) to demarcate them from *Burkholderia sensu stricto*, ** which comprises environmental, human clinical, and phytopathogenic species (the alleged bad ones) ^72^. In this study, the percentage guanine plus cytosine content and conserved indels in whole genome sequences of some 25 formally named *Burkholderia* species and several unclassified strains were studied. Species belonging to the *Burkholderia sensu stricto* clade were characterized by a percentage guanine plus cytosine content of 65 to 69% and shared six conserved sequence indels, while all other *Burkholderia* strains examined had a percentage guanine plus cytosine content of 61 to 65% and shared two conserved sequence indels. The phylogenetic heterogeneity among the remaining *Burkholderia* species as revealed by 16S rRNA-based divergence and by differences in the distribution of 22 additional conserved sequence indels was ignored, as the authors proposed reclassifying all remaining *Burkholderia* species into a single novel genus, *Paraburkholderia* ^72^. These novel names were subsequently validated ^75^ and now have formal standing in bacterial nomenclature. The scientific community may adopt these novel names or not. Authors who are convinced that these name changes are ill founded can continue to work with the original species names, as all these were validly published.

A recent study employed comparative genomics to assess the pathogenic potential of environmental strains on the basis of the presence or absence of known virulence factors ^73^. This bioinformatic study clearly showed that many virulence factors, including the type III, IV, and VI secretion systems, are mostly found in representatives of the *Burkholderia sensu stricto* clade while they are often absent in strains of the *Paraburkholderia* clade. The authors also show that *Paraburkholderia* strains exhibit no virulence in a *Caenorhabditis elegans* infection model. While these are valuable approaches, they also have their caveats. Many virulence factors of *Burkholderia* species have been shown to be host specific, and there is little correlation between the different infection models commonly used, e.g. *C. elegans*, *Galleria mellonella*, and *Drosophila melanogaster*. This probably reflects the need for *Burkholderia* strains to compete for survival in diverse habitats such as soil, plants, insects, and mammalian hosts. Only very few universal virulence factors could be identified in *B. cenocepacia* (namely quorum sensing, siderophore production, and lipopolysaccharide biosynthesis) and therefore extrapolations from non-mammalian infection models to mammalian infections, particularly to chronic CF lung infections, must be made with caution ^76, 77^. For example, most *Burkholderia multivorans* strains show no virulence in a *C. elegans* or *G. mellonella* infection model ^78, 79^, although most virulence factors that were suggested to be indicative for pathogenic *Burkholderia* species could be identified in this Bcc species ^73^. Yet *B. multivorans* (along with *B. cenocepacia*) is one of the predominant *Burkholderia* species infecting people with CF ^58, 80^. On the other hand, *B. cenocepacia* strain H111 ^81^, which is closely related to strains of the epidemic ET12 lineage (e.g. J2315 and K56-2), did not cause acute symptoms in the infected CF patient from whom it was isolated and was cleared after a 6-month co-infection period with *Pseudomonas aeruginosa* ^82^, while infections with strains of the ET12 lineage have resulted in high mortality among patients ^58, 83^. In contrast to its clinical impact, strain H111 shows a similar level of pathogenicity in the *G. mellonella* and *C. elegans* infection models to K56-2 (an ET12 lineage strain) and both strains are much more virulent in these models than J2315 (another ET12 lineage strain) ^77^.

These examples strongly suggest that neither the presence of virulence genes in a strain nor acute virulence as assessed in routinely used non-mammalian infection models is an absolutely reliable predictor of clinical prevalence or outcome in CF patients. The taxonomic position of a strain is also not an unambiguous indicator for its pathogenic potential and thus decisions on the industrial or biotechnological use of a *Burkholderia* strain can be made only on a case-by-case basis after careful molecular and phenotypic characterization of the strain. On the comparative genomics side, it will be interesting to see whether the co-occurrence of certain genes may be a suitable indicator of the phenotypic potential of a strain, as has recently been proposed in the case of plant-growth-promoting bacteria ^84^.

## The use of *Burkholderia* as biocontrol agents

Although endophytic or nitrogen-fixing *Burkholderia* strains show great promise as agents for plant growth promotion and bioremediation, it should be kept in mind that in terms of biocontrol applications the most outstanding property of *Burkholderia* strains is the production of various compounds with potent antifungal activity ^14, 85^. In fact, several Bcc strains have been registered by the United States Environmental Protection Agency (EPA) for use as biocontrol agents against phytopathogenic fungi, including Deny®, Blue Circle®, and Intercept®, in the 1990s. However, after risk assessment, these products were withdrawn from the market and the EPA placed a moratorium on the registration of products containing Bcc species ( https://www.gpo.gov/fdsys/pkg/FR-2004-09-29/pdf/04-21695.pdf). Would it be possible to replace these Bcc-based biocontrol agents with strains of the *Paraburkholderia* lineage? Literature research, genome mining, and experimental evidence ( Figure 1) have revealed that only three species of the *Paraburkholderia* cluster, namely *Burkholderia phenazinium*, *Burkholderia megapolitana,* and *Burkholderia bryophila,* all of which have been isolated from mosses ^86^, show antifungal activity. In contrast, most strains of the Bcc and many of the human and plant pathogenic species produce antifungal compounds ^85^. Given that most antifungal agents exhibit more general toxic effects in eukaryotic organisms, these compounds may contribute to the virulence of a strain. *B. phenazinium* was reported to produce the phenazine iodinin ^87^, which exhibits not only high anti-microbial but also cytotoxic activity. While iodinin may be valuable for clinical purposes, as it is potent against leukemia cell lines ^88^, it may not be useful for biocontrol applications. To our knowledge, the antifungal compounds produced by *B. megapolitana* and *B. bryophila* have not been identified nor has their pathogenic potential been evaluated in an infection model. In conclusion, while many Bcc strains have been demonstrated to exhibit excellent biocontrol activities, there are only very few *Paraburkholderia* strains that are potentially useful for biocontrol purposes.

## Is there a safe *Burkholderia* strain?

Given the lack of reported cases in the literature, many strains of the *Paraburkholderia* lineage seem unlikely to cause infections in humans and therefore could be considered for agricultural applications. The same may also apply to some strains of the *Burkholderia* lineage, as has recently been suggested for the Bcc strain *Burkholderia contaminans* MS14, which was found to possess multiple antimicrobial biosynthesis genes but not major genetic loci required for pathogenesis ^89^. While the phylogenetic status of a strain may be helpful as a first approximation of the pathogenic potential of a strain, it is clear that the *Paraburkholderia* lineage contains some pathogenic strains and that several Bcc strains exhibit good biocontrol properties and attenuated virulence. Hence, independent of a strain’s phylogenetic status, a thorough characterization of a strain will be required before it can be considered safe. It will be important to use well-established infection models such as the mouse model ^90^ for the assessment of the potential pathogenicity of a strain and to carefully examine whether related strains have been isolated from infected humans. Likewise, the biocontrol activity of the strain has to be tested in field trials. It is also worth noting that several species of the *Paraburkholderia* clade, including the well-investigated endophyte *B. phytofirmans*, are unable to grow at 37°C (in contrast to *Burkholderia sensu stricto* species), a property that is considered to be essential to infect and colonize humans. The capability to grow at 37°C has recently been proposed as a simple means to differentiate between pathogenic and non-pathogenic *Stenotrophomonas maltophilia* and *Stenotrophomonas rhizophila* isolates ^91^. Representatives of the latter species have therefore been suggested to provide an alternative to biotechnological applications without posing any risk to human health ^92^. An important line of future research will therefore be to assess the pathogenicity of environmental strains in suitable infection models, particularly using a mammalian host at 37°C, and ideally in multispecies infection scenarios, which may more accurately reflect the genuine clinical situation.
